# Perspectives and State of the Art of Membrane Separation Technology as a Key Element in the Development of Hydrogen Economy

**DOI:** 10.3390/membranes14110228

**Published:** 2024-10-30

**Authors:** M. Olga Guerrero-Pérez

**Affiliations:** Departamento de Ingeniería Química, Universidad de Málaga, E29071 Málaga, Spain; oguerrero@uma.es

**Keywords:** blue hydrogen, green hydrogen, white hydrogen, gas separation, membranes, hydrogen economy, proton-exchange membrane fuel cell, hydrogen energy, proton-exchange membrane (PEM)

## Abstract

Due to the objectives established by the European Union and other countries, hydrogen production will be a key technology in the coming decades. There are several starting materials and procedures for its production. All methods have advantages and disadvantages, and the improvements in their performance and decreases in operational costs will be decisive in determining which of them is implemented. For all cases, including for the storage and transport of hydrogen, membranes determine the performance of the process, as well as the operational costs. The present contribution summarizes the most recent membrane technologies for the main methods of hydrogen production, including the challenges to overcome in each case.

## 1. Introduction

Hydrogen is an energy carrier that can be produced using many different processes and starting materials. A color terminology that denotes these hydrogen production methods has been established [1]. Table 1 briefly describes the main methods with their advantages and disadvantages. According to this terminology, when hydrogen is produced through water electrolysis, the energy for which is produced using renewable sources (mainly solar), the term “green hydrogen” is used. The European Union established the REPowerEU [2] plan in 2022, in which renewable hydrogen is considered as a substitute for fossil-derived fuels, with the objective of decreasing dependence on Russian fossil fuel imports and accelerating the decarbonization of the European economy. Since most renewable energy sources are intermittent and geographically constrained, there are temporal and spatial gaps between energy availability and consumption by end-users [3]. The transformation of renewable energy into hydrogen, which can be transported, seems to be a solution to this problem, although it could concentrate the production of solar energy in determined areas. In any case, this is quite an ambitious objective, since natural gas and coal are the primary sources used in hydrogen production [4] through coal gasification or steam methane reformation (called grey or black hydrogen). These processes are much less expensive than green hydrogen production and do not require large amounts of water, although natural gas and coal are required as starting materials. In this process, CO_2_ emissions are produced, and the ability to capture and store them makes this an interesting option; in this case, the term blue hydrogen is used (Table 1). Some companies such as Shell and BP are betting on processes based on blue hydrogen technologies through the steam methane reformation of natural gas with carbon capture and storage (CCS) technologies, with the aim of limiting CO_2_ emissions; for example, Shell recently announced contracts for blue hydrogen projects in Oman [5] and Ohio [6], whereas BP will increase its focus on blue H_2_ in this decade [7]. Green hydrogen will be an option in the long run, but it is not currently competitive with blue hydrogen. Thus, green hydrogen is a good option that does not require natural gas, but it is expensive in comparison with other technologies such as blue hydrogen; in addition, it requires large amounts of water and land (for solar panels). In this context, several research groups are focusing on the design of catalysts for seawater electrolysis, although this process is still far from commercial implementation [8,9]. In addition, large deposits of natural hydrogen (white hydrogen) are being discovered, which is a market disruptor, since they are a relatively cheap source of hydrogen. According to recent data (April 2024) from the World Economic Forum [10], the production costs for green hydrogen technologies are in the USD 6–12/Kg range, whereas white hydrogen can be delivered for around USD 1/Kg. We are, therefore, in a situation regarding hydrogen technologies similar to what happened more than a century ago with the black gold rush. No one doubts that decarbonization is necessary, both to avoid CO_2_ emissions and because fossil fuel resources are limited, or that hydrogen will be the most common fuel in a few decades, since its combustion produces only water and is completely clean. However, there are different methods to produce it from different raw materials. There is also the option of extracting it naturally, although it is not known for sure how much hydrogen there is in the subsoil; this is being explored right now. Everything indicates that the technologies that will be used in the next decade will be green, blue, and white, and the production costs will be decisive in seeing which of the three options prevails in the market. In this context, gas separation membranes [11], together with electrocatalysts, will be decisive. In all processes, membranes will be necessary and will determine their efficiency and effectiveness.

In blue hydrogen production, a light hydrocarbon is used as a starting material (i.e., methane, naphtha, natural gas), mainly using a reforming process (SMR), partial oxidation (POM), or a combination of both (ATR technologies). Thus, there are different configurations, and CCS can be carried out pre- or post-combustion. Precombustion CCS technologies refer to CO_2_ removal from the starting syngas, whereas post-combustion CCS refers to removal from the exhaust gas. There are different processes for separating CO_2_ [12], such as absorption (e.g., amine scrubbing) or cryogenic processes [13]. However, these processes are energy-intensive and require the use of corrosive and environmentally harmful absorbents. Because of this, membranes [14,15] play a very important role in industrial-scale H_2_/CO_2_ separation due to their economic feasibility, easy operation, cost maintenance, term durability, and design flexibility (Table 2).

Regardless of the method used for hydrogen production, purification is required for almost all applications; for example, fuel cells and ammonia synthesis require high-purity hydrogen gas to operate efficiently and reliably, since impurities, even at a low concentration, can affect their processes [15]. Therefore, there are currently several processes and raw materials involved in hydrogen production processes; in all of them, membranes are decisive in improving efficiency and reducing costs. Therefore, it is the objective of this work to concisely analyze the state-of-the-art membrane technologies that are decisive in hydrogen separation and purification processes and the most recent technologies. Please note that this is not an exhaustive review on membrane technologies for H_2_ purification.

## 2. Blue Hydrogen: CO_2_/H_2_ Separation Membranes

**Figure 1:**
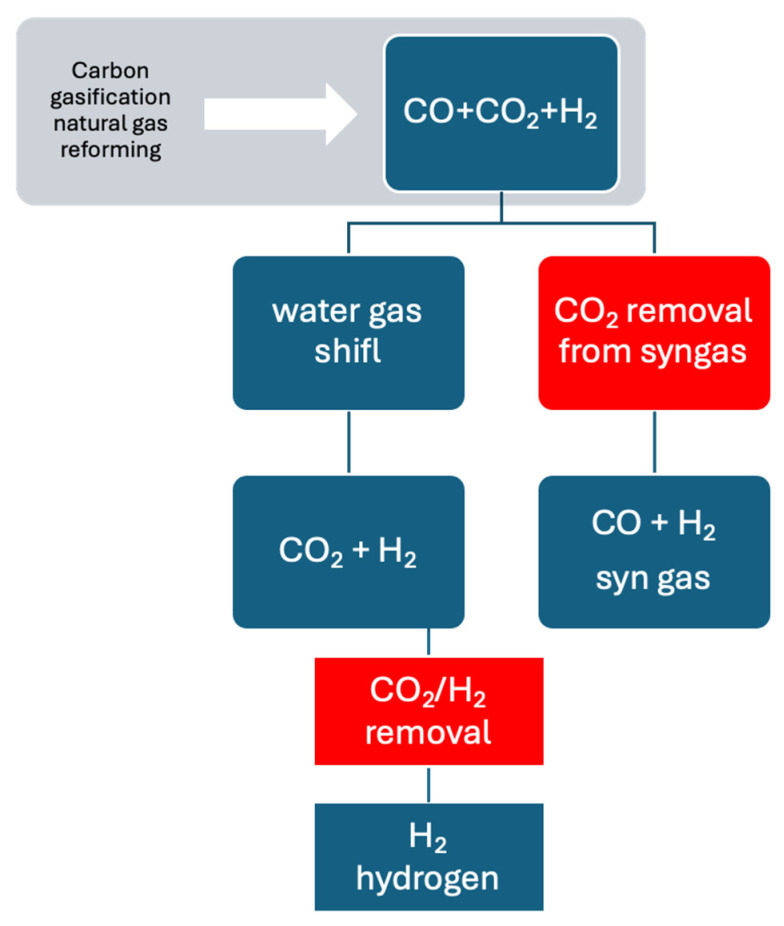
Scheme of the process for hydrogen and synthesis gas via carbon gasification or natural gas reformation with precombustion CCS.

As already introduced, hydrogen is commonly generated through the steam reformation of methane, and this process mainly yields a CO and H_2_ mixture. Through the water–gas shift reaction, CO in this mixture is transformed into CO_2_ (Figure 1). CO_2_ can be removed from the synthesized gas in order to generate a synthesized gas stream that should find several applications in the chemical industry. The Membrane Technology Research company has developed a commercially available membrane system called Polaris that is able to separate CO_2_ from syngas [17]. For the production of a purified H_2_ stream, it is necessary to separate the CO_2_/H_2_ mixture.

Thus, commercially available CO_2_/H_2_ separation processes are necessary for the development of blue hydrogen technology. Membranes play an important role in this separation, since other technologies such as absorption require the use of amine-based solvents, which are corrosive, expensive, and energy-intensive (Table 3). Membrane technology actually presents some limitations (Table 3) in commercial implementation, but there is an important research effort to overcome these limitations, since it is the most promising technology and present several advantages, such as flexibility, low energy requirements, and low production costs.

The requirements that have been established as an industrial target for hydrogen production include membranes with H_2_ permeance higher than 1000 GPU and CO_2_/H_2_ selectivity higher than 60 [18]. The membranes used for CO_2_/H_2_ can be inorganic (metal–organic frameworks, metallic, oxides, ceramics) or organic (polymers, celluloses). Several mechanisms are involved in the membrane separation process, with the most important being solution diffusion (defined by the gas permeability) and molecular sieving (defined by the selectivity). The gas permeability of a membrane is defined as the ease with which atoms or molecules can pass through the membrane in the presence of a pressure differential; this can refer to the membrane thickness and area, the pressure difference, or the permeation flow rate, modeled by equations such as Fick’s law. On the other hand, the selectivity refers to the membrane’s permeability to single atoms/molecules [19,20]. The permeability coefficient is defined as the product of the membrane thickness and the gas flux divided by the pressure gradient. The membrane selectivity is defined by the ratio of the permeability of components to the membrane thickness (α_A/B_ = P_A_/P_B_, with P_A_ and P_B_ being the permeability factors of components A and B in a binary mixture) [21]. When the membrane pore size and the kinetic diameter of the molecules that penetrate (Table 2) are similar, the gas transport is dominated by the molecular sieving mechanism. The graph of the selectivity versus the permeability is known as the “Robeson Plot”, and it is commonly used for showing membrane performance [22]. These mechanisms mainly include thermodynamic and kinetic contributions; depending on the type of material and the process conditions, each of them can be dominant [23]. The goal when designing a CO_2_/H_2_ membrane is to achieve both high selectivity and high permeability, in addition to a long lifetime and high robustness.

**Figure 2:**
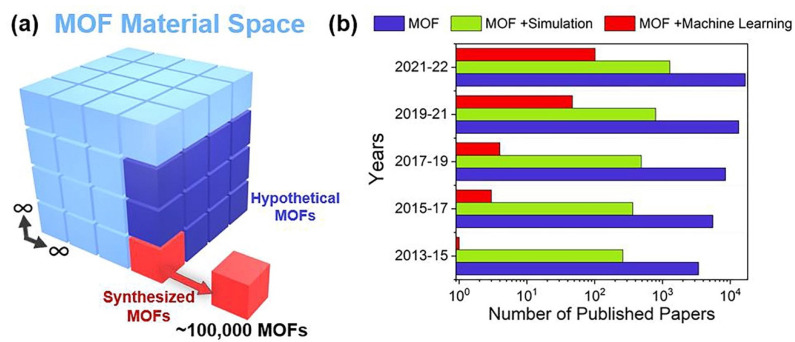
(**a**) The large cube represents the MOF space with an almost infinite number of materials, including synthesized and hypothetical MOFs. (**b**) The number of published papers having the keywords (i) “MOF”, (ii) “MOF” and “Simulation”, and (iii) “MOF” and “Machine Learning” in their titles and abstracts. Data were retrieved from the Web of Science on 17 October 2022. Reproduced with permission from [35].

Inorganic membranes present several advantages since they present high durability and stability in a wide range of conditions [24,25]. They can be dense membranes (mainly ceramic and metallic membranes) or porous membranes (such as zeolite, MOF, and carbon-based membranes). Zeolites have been widely investigated as promising materials for CO_2_/H_2_ separation since they exhibit high selectivity [26] and can be synthetized as hollow fibers [27,28,29] using scalable methods, such as electrospinning [30], which reduce the membrane cost significantly and increase the packing density of the membrane module [31]. In addition, their properties can be modulated through functionalization, for example, with amines, to increase selectivity at low pressures. In a recent study, Karanikolos and coworkers [32] proposed this approach. These studies, based on zeolite materials, are promising. MOF (metal–organic framework) membranes are also gaining attention due to their tunable properties (pore size, chemical surface groups, surface area, etc.), which can be modulated through the use of different metals and organic linkers. A recent study by Keskin et al. reviewed the use of MOF membranes for CO_2_ capture [33], showing how these membranes can efficiently separate CO_2_/H_2_ mixtures, especially more recently developed membranes (glass, ultrathin, and hybrid). In this case, the MOF materials are designed with functional groups able to alter their affinity towards desired/undesired species (such as impurities like H_2_O or H_2_S that may be present) and tailor the size and shape of the pores [30]. Computational modelling is helping to guide the experimental design of membranes with the desired properties [34], since hypothetical MOF materials can be computationally generated, exploring several combinations of components. To gain an understanding of the status of MOF materials research, Keskin et al. performed a bibliographic study, shown in Figure 1 [35]. Figure 2a presents all the hypothetical MOF materials that could be prepared, underlining (in red) those that have already been synthetized (about 100,000, whose crystallographic information files have been deposited into the Cambridge Structural Database); those that have been computationally generated are shown in dark blue. Figure 2b illustrates the number of published papers related to these materials, which increases every year due to the high potential of these materials; however, it should be noted that the percentage of studies related to simulation and using machine learning is increasing. Thus, the potential of MOF materials, including their use for the preparation of membranes for CO_2_/H_2_ separation, is a hot topic that is still developing, although promising results have already been reported [36,37].

**Figure 3:**
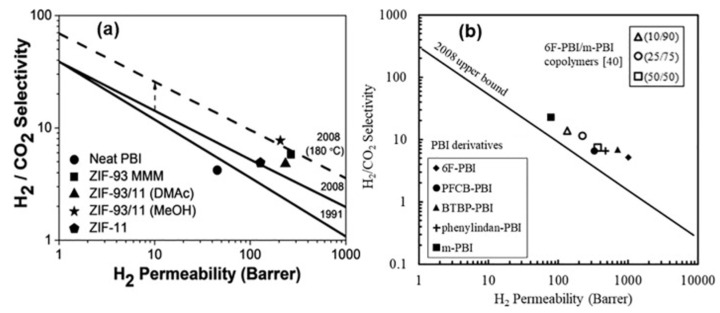
(**a**) H_2_/CO_2_ gas separation performance of a polymer with 20 wt% loaded MOF material [37]). The Robeson upper bounds are denoted with continuous lines, while the estimated upper bound at 180 °C is denoted with a dashed line. (**b**) Performance comparison of different derivatives and random copolymer membranes for the separation of H_2_/CO_2_ [38]. Reproduced with permission from [39].

These inorganic materials can be combined with a polymeric material to prepare mixed-matrix membranes (MMMs) that present the advantages of a polymer matrix (mechanical and tensile strength) with organic/inorganic material. Usually, zeolites, MOFs, and carbons are used as fillers for the fabrication of these MMMs. Figure 3 illustrates the separation performance of several MMMs in which polymers were loaded with different amounts of MOF materials. Figure 3a illustrates how the H_2_/CO_2_ separation performance increased with respect to both the bare polymer (marked with a circle) and the bare MOF material (marked with a pentagon); Figure 3b shows the hydrogen permeability for several MMMs.

## 3. Green Hydrogen: Membranes for Electrolyzers

In the case of green hydrogen production, H_2_ streams are produced through water electrolysis that is carried out in an electrolyzer. As explained in the introduction, one of the main challenges in green hydrogen technology is to reduce its operational costs with respect to blue hydrogen. Although fuel cell technology and water electrolyzers have made significant progress in the past few decades, their commercialization still faces many challenges, such as the short lifespan of fuel cells. Performance degradation is caused by a variety of complex factors, including the degradation of electrode materials, a loss of catalysts, mechanical damage to the membrane electrode assembly, and fluctuations in the operating conditions [40]. In this sense, membranes are a very relevant cost associated with fuel cell and electrolyzer performance, since they are responsible for the lifetime, power density, and efficiency of these devices.

**Figure 4:**
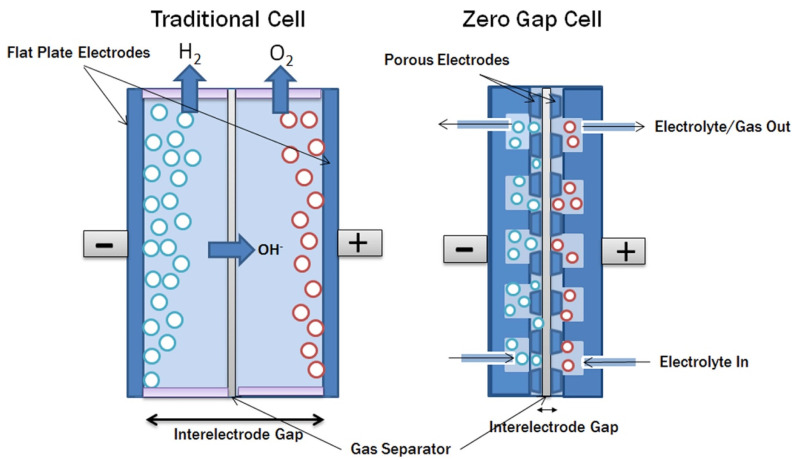
Schematic of the zero-gap cell design versus the traditional cell design. Reproduced with permission from [54].

There are different electrolysis technologies that require different types of membranes; however, in all cases, membranes play an essential role, as they are required to facilitate ion transfer and to act as a barrier to separate anode and cathode gases [41,42]. Table 4 summarizes the schematic data regarding the three main technologies: PEMWE (proton-exchange membrane water electrolysis), AEMWE (anion-exchange membrane water electrolysis), and BPMWE (bipolar membrane water electrolysis). PEMWE electrolyzers are commercially available, and this technology is well known. There are several commercial membranes based on different polymeric materials, such as perfluorosulfocic acid (PFSA) or polytetrafluoroethylene (PTFE). Since the synthesis of these polymers includes toxic intermediates, there is a significant research effort to develop low-cost non-fluorinated polymers [43] that present additional advantages, such as biodegradability and high thermal stability [43]. Despite the high number of commercial membrane technologies available for electrolyzers and fuel cells, there is still a need to increase their durability and performance and to lower their cost. In the case of AEMWE technology, there are also commercially available membranes that are stable under high pH based on hydrocarbon polymers, such as polyolefins [44], polyfluorenes [45], polyphenylenes [46], polyarylene piperidinium [47], or imidazolium [48]. The durability of these membranes is frequently limited by phenyl [49] and polystyrene [50] electrochemical degradation. Another important issue for these membranes is the interfacial resistance between the membrane and the electrode, which causes low current density. To overcome these problems, several solutions have been proposed; these include the zero-gap cell design (Figure 4), which comprises porous electrodes on each side of the membrane [51,52,53], facilitating a reduction in cell resistance to the gas bubbles, as well as to the electrolyte.

Bipolar membranes [55,56] are composed of cation- and anion-exchange layers that selectively transport H^+^ and OH^−^ and are used in BPMWE electrolyzers, integrating alkaline and acid half reactions at different pHs (Table 4). In this manner, both OER (oxygen evolution reaction) and HER (hydrogen evolution reaction) can be carried out at their optimal pH. Alkaline OER can be catalyzed using low-cost and abundant elements, such as Fe or Ni, whereas the acid OER uses in PEMWE electrolyzers requires the use of noble metals. Although there have been advances in the development of noble metal-free electrocatalysts for acid OER, they are not currently available [57]. In addition to avoiding the use of noble metals, another advantage is that BPMWE technologies allow for the use of impure water, since the alkaline pH at the anode limits the Cl^−^ oxidation to corrosive byproducts [58,59]. Thus, this technology could allow for the development of direct seawater electrolysis, which represents a promising solution regarding the main disadvantages associated with green H_2_ production, namely, that fresh water is required [60].

## 4. White Hydrogen and Transportation: H_2_/CH_4_ Separation

**Figure 5:**
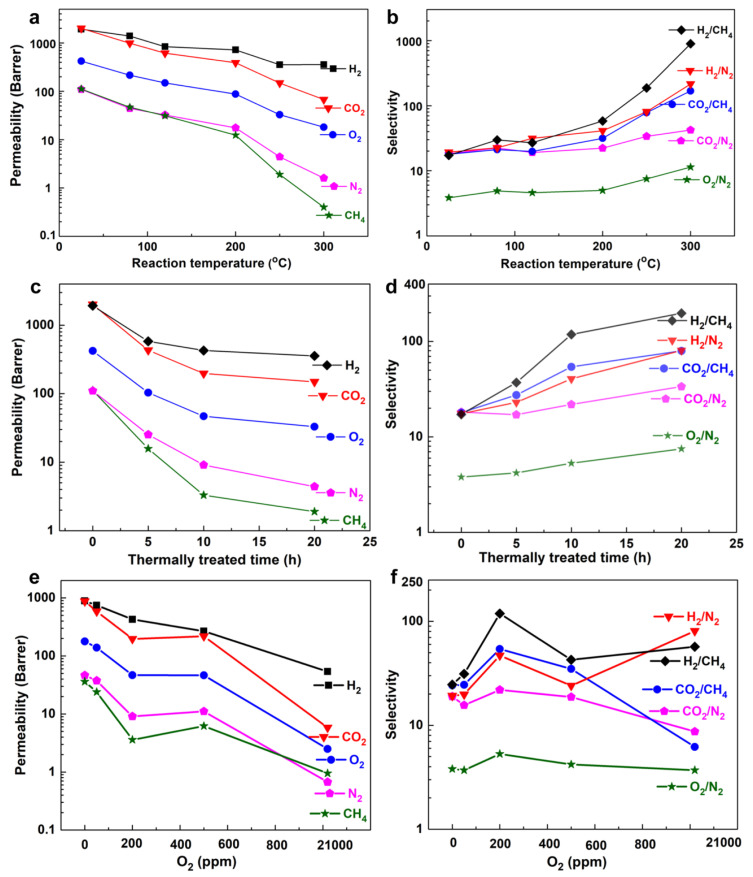
(**a**) Gas permeability and (**b**) selectivity as a function of reaction temperature for several membranes enabled by multi-covalent crosslinking of microporous polymer blends, thermally treated at the indicated temperature for 10–20 h under 200 ppm of O_2_. (**c**) Gas permeability and (**d**) selectivity for crosslinked membranes treated at 250 °C, versus treated time. (**e**) Gas permeability and (**f**) selectivity as a function of O_2_ concentration for crosslinked membranes treated at 250 °C for 10 h. Reproduced with permission from [65].

The separation of methane from hydrogen is quite relevant to refinery off-gas processing [61], and it is required in the case of white hydrogen, since natural hydrogen is usually found as a mixture with methane. In addition, an option for hydrogen transportation is the injection of 6–10% hydrogen in natural gas pipelines. This H_2_/CH_4_ gas mixture (hydrogen-enriched natural gas) can be utilized directly for power generation, or the hydrogen and methane can be purified in place for the desired end use. Thus, there are several applications in which H_2_/CH_4_ separation is required, and membrane technology is one of the most useful and economically viable options for this. The materials required for the preparation of H_2_/CH_4_ separation membranes are the same as those discussed for H_2_/CO_2_ separation: inorganic materials, such as zeolites, MOF [62] carbons, polymers, and MMMs [63]. According to the kinetic diameter (Table 4), the permeability of CO_2_ is higher than that of CH_4_; subsequently, the selectivity for H_2_/CH_4_ is higher than for H_2_/CO_2_ in most types of membranes (Figure 5) [64,65]. Although a large number of commercial membranes are available for this separation, there are still some challenges to overcome [63], since, for most of the actual applications, they have to be used in harsh conditions (high temperature and pressure and in the presence of impurities such as water vapor), and it is necessary to extend the lifetime of the membranes and their mechanical properties in these environments. In addition, and in order to achieve the performance that the applications require, the microstructure of the membranes should be well controlled, and the thickness has to be reduced in order to build membrane modules.

## 5. Conclusions and Perspectives

The demand for hydrogen as a fuel is increasing due to the objectives of the European Union and other countries to achieve decarbonization. There are different raw materials and processes used to obtain hydrogen while avoiding the use of fossil fuel resources, and all of them present advantages and disadvantages (Table 5), as has been explored. However, in all cases, including for the storage and transport of hydrogen, membranes are the key to achieving lower-cost processes and improving their efficiency.

In the case of blue hydrogen and white hydrogen technologies, H_2_/CH_4_ and H_2_/CO_2_ membrane separations are required. Although several commercial membranes are available, there are still some challenges to overcome (Table 5) in order to improve the selectivity, improve the mechanical properties, and decrease the costs. For green hydrogen technology, membranes are a key component of all electrolyzer varieties, as exposed in this study. In addition, the most recent electrolyzer technology, based on bipolar membranes, is crucial for the development of direct seawater electrolysis.

## Figures and Tables

**Table 1:** Main H_2_ production methods with the most important pros and cons associated with them. Note that, for the sake of simplicity and to focus on this study’s contents, other methods, such as those in which the energy for the water electrolysis is provided by nuclear energy, are not included in this table.

	Black	Grey	Blue	Green	White
	Gasification of coal	Methane steam reforming	Produced from fossil fuels (i.e., grey or black), where CO_2_ is captured and stored	Produced via electrolysis of water using electricity from renewable sources (wind or solar)	Naturally produced in the Earth’s crust
Cons	CO_2_ emissions. Fossil fuel source required.	CO_2_ emissions. Fossil fuel source required.	Fossil fuel source required.	High production costs. Large amount of freshwater required. Solar energy requires the use of large surfaces of land or sea.	Cheap (it does not require energy conversion/manufacturing processes). It is quite a new technology, and geological surveys are required.
Pros	Low production costs. Available raw material.	Low production costs.	Low production costs in comparison with green hydrogen. Low CO_2_ emissions.	Renewable Zero CO_2_ emissions.	May be renewable. Low environmental impact.

**Table 2:** Physical properties of hydrogen, methane, and CO_2_ gas molecules [16].

	Molecular Weight (g/mol)	Kinetic Diameter (A)	Critical Volume (cm^3^/mole)	Critical Temperature (K)
H_2_	2	2.89	65.1	33.24
CH_4_	16	3.80	99.2	191.05
CO_2_	44	3.30	93.9	304.21

**Table 3:** Main limitations and advantages of the current technology processes for CO_2_/H_2_ separation.

	Absorption	Adsorption	Distillation	Membranes
Advantages	Commercially available technology with high CO_2_ removal rates.	High number of adsorbents available with high adsorption capacity.	Available for high CO_2_ concentrations.	Low capital and operation costs. Flexibility. Simple operation.
Limitations	Use of corrosive absorbents, energy- and cost-intensive processes, low absorption capacity.	The advantages that adsorption presents, such as decreased pressure requirements and a sensitivity to impurities, do not seem to be sufficiently profitable to replace current absorption processes with it.	High operation costs.	They are more expensive than absorption and adsorption and may present low flux, high fouling, and some instability under high T and P working conditions.

**Table 4:** Reactions involved in PEMWE, AEMWE, and BPMWE technologies.

	PEMWE	AEMWE	BPMWE
Reactions occurring at the anode and cathode	**2H^+^ + 2e^−^ → H_2_** H_2_O → 2H^+^ + 0.5O_2_ + 2e^−^	2H_2_O + 2e^−^ → 2OH^−^ + H_2_ **2OH^−^ → H_2_O + 0.5O_2_ + 2e^−^**	**2OH^−^** **→ H_2_O + 0.5O_2_ + 2e^−^** **2H^+^ + 2e^−^** **→ H_2_**
	HER acid OER acid	HER alkaline OER alkaline	HER acid OER alkaline
Membrane selective to:	Protons (H^+^)	Anions (OH^−^)	Bipolar membrane (OH^−^ and H^+^)

**Table 5:** Summary of the main pros and cons and state of the art of membranes for H_2_ production with blue and green technologies.

	Blue H_2_	Green H_2_
Most promising membrane type	Mixed-matrix membranes (MMMs).	Bipolar membranes.
Main advantages of H_2_ production technology	Low costs. CO_2_ emissions are avoided. Captured CO_2_ can be used as a raw material.	Renewable.
Main disadvantages of membrane technology	Low flux, high fouling, instability under high T and P.	Poor lifetime, power density, and efficiency.
Main problems to be solved	Absorption methods are less expensive. It is necessary to decrease the membrane costs to compete with them and avoid the use of dangerous and corrosive sorbents.	Green H_2_ technology consumes a large amount of fresh water. It is necessary to develop electrolysis technology compatible with sea water; in this context, bipolar technologies are promising. For large-scale production, it will be desirable to use the O_2_ produced as a by-product.

## Data Availability

No new data were created or analyzed in this study. Data sharing is not applicable to this article.
